# Modern Sociology

**Published:** 1902-08-23

**Authors:** 


### Modern Sociology.

### ELEMENTARY TEACHING: AN OPENING FOR EDUCATED WOMEN.

The notion that women who can do nothing else for a living had better turn governess is dying out. Not so many years ago it was almost the only genteel occupation for ladies who found themselves cast on their own resources; they either "entered a family," or "opened a little school." Some of these little schools are not quite dead yet, and a special inquiry made recently by the Board of Education brought to light some pitiful tales of inadequacy struggling to keep abreast with the march of modern requirements.

Happily for everyone concerned, and not least for the teacher herself, we are changing all that. Fifty years have seen a great advance; girls no longer drift in such numbers into a profession for which they are neither fitted nor trained. The best schools will not have them, and the inferior ones are doomed. It is as a profession that women now take up the work of teaching, and a specific training is an absolute necessity. "Our schools," says a recent writer, 'are no longer staffed by women who have begun their work in life driven to it by necessity or disappointment." Thanks to the women who "at the risk of great unpopularity and much social loss fought the battles by which the doors were opened," the necessary training for this and other professions is possible.

Although the high school, perhaps, offers the most obvious opening for the highly trained teacher, in elementary schools there is a new and interesting field for educated women of the "higher classes." As a career for high school girls and others who have had a secondary education, it has not yet received much attention, but many are already at work, and many more are training with those who until now have occupied the field. No less an authority than Sir Joshua Fitch has said that it is not desirable that all the teachers in the schools aided and inspected by the Board of Education should have entered the profession by exactly the same path. "Variety of type among the teachers is essential in so comprehensive a national system as our own. And any measures which will introduce into the ranks of certificated mistresses well-instructed women, who have not been pupil teachers, but have received their education in higher or intermediate schools, and who have been attracted to the work by a liking for it, and by a sense of personal fitness, will not only supply the State with a greater variety of skilled agents, qualified for different kinds of work, but will also open to many gentlewomen new possibilities of honour and public usefulness, and of an interesting and happy life."

The advantages which it offers may be briefly stated thus:—(1) A girl's education can be carried on at home or at school until the age of 16 or 17. (2) The training is comparatively short and inexpensive. (3) It is almost the only profession open to women which is not overstocked, and Sir George Kekewich remarks that "having the certificate of an elementary teacher in your pocket means certain bread-and-butter whenever you like to use it." If marriage or other circumstances cause the work to be given up after a time, the certificate holds good at any future period. (4) It carries with it a small pension in the case of old age or incapacitation; and compulsory payment on the military system being deducted from the salaries, the amount due can be claimed whenever the work is given up. (5) For college-trained and certificated teachers, besides posts in elementary schools as head and assistant mistresses, there are those as teachers and lecturers in day and residential training colleges and pupil-teacher centres; good offers come sometimes from the Colonies, and at the present time there are openings in South Africa. (6) Salaries vary under different boards, but they average from £70 to £90 for certificated assistant teachers, and from £90 to £150 for head mistresses. Assistant teachers, non-certificated, under Articles 50 and 51 of the Day School "Code" vary from £50 to £90: these may, if approved by the inspector, take charge of schools (with an average of not more than 40 children) under Article 82. There still remains the teacher under Article 68, whose only qualification is that she is 18 and approved by the inspector: she receives from £20 to £40. (7) It is a national work; and, as Miss Lucy Soulsby, late head mistress of Oxford High School, says, "the noblest women of the highest culture may feel honoured by taking it up when the immense power over the future rulers of the country which the elementary teacher possesses is taken into consideration."

The normal course of preparation is the pupil teachership of one, two, three, or four years in an elementary school, followed by the King's Scholarship examination and two years at a training college, or an assistantship in an elementary school until the certificate is passed: and although it is desirable that the young mistress should bring to the work the wider culture of a high school, it is essential, if she is to compete with teachers thus trained, that she should be as perfect as possible in all technical details. Some high schools have already formed classes for the study of the technical side of teaching. The drawback, however, seems to lie in the absence of all acquaintance with the practical work of an elementary school. Lectures and study in the class-room of a high school may bring success in examination, but not turn out a capable teacher.

It is in order to supply this deficiency that one of the Diocesan Training Colleges—that at Salisbury—has a hostel attached, more particularly for girls of the class we are considering. The pupils live at the hostel, where they receive a thorough and special education, and they also attend an elementary school for observation and actual practice. This is essential to success in work which will afterwards be carried on under very different conditions from those obtaining in a school where the pupils are of the "secondary" type. Special preparation is given for the King's Scholarship, and others who have passed equivalent examinations are received for periods varying to suit each case, for the strengthening of weak subjects, and the teaching experience which is gained under efficient guidance in the practising schools, and older women can be admitted for the "partial" training which will qualify them to teach under Art. 50, which offers both employment and salary, and which will help to meet a great demand. "The difficulties of the position socially," says the lady superintendent of the hostel, "are even now fast disappearing. The presence of a lady at the village school house would be welcomed by many a rector's wife, while in the large towns suitable introductions can always be found. A girl can not only share rooms with a brother or sister who is working for a living too, but can make a home sometimes for a mother. The intercourse with those already teaching has been found to be both pleasant and helpful. The drawbacks and drudgery of the profession are far less than in many an occupation taken up nowadays, and it offers the impetus which some lack, of a strong human element of interest."

Seventeen First Classes in the following examinations have been gained in the past three years by the hostel students:—Queen's Scholarship, Archbishop's Examination in Religious Knowledge, and Science (South Kensington).

